# Belgian Law against Tuberculosis in Cattle

**Published:** 1896-04

**Authors:** 


BELGIAN LAW OF OCTOBER 30, 1895, AGAINST TUBERCULOSIS IN CATTLE. This law, consisting of thirty-four articles, goes into effect January 1, 1896. The following are the principal points:

Every animal coming from a foreign land must on importation be provided with an indelible mark. In case it, on account of the existence of clinical symptoms, is considered as suffering from or is suspected of tuberculosis, it shall be immediately sent back to the country from which it was imported. If this is not complied with, the animal must be dispatched within three days.

Importation of cattle is forbidden from any country in which tuberculosis exists; if it is decided not to insist on this regulation, then the cattle which are to be imported must be subjected to a tuberculin-test.

Importation of tuberculin or the transportation of the same within the land and inoculation with the same cannot take place without the authorization of the Minister of Agriculture and Public Works.

Whenever an animal upon slaughter shows signs of having suffered from tuberculosis, the owner must make declaration of the same; and the cattle from the same stable are to be declared suspected. Every veterinary surgeon who recognizes tuberculosis in a living or dead animal must report this at once to the veterinary surgeon of the district. Cattle that, on account of the existence of clinical symptoms, are found to be suffering from tuberculosis, and in like manner those which being suspected have reacted upon the test, are to be killed. The cattle which are standing in the same stable or yard cannot be sold for food purposes unless they have been inoculated with tuberculin and have *not* reacted upon it. These inoculations take place at the expense of the State.

Every cattle-owner can receive authority to subject his cattle to the tuberculin-test; if one animal reacts, it must be dispatched immediately, and the test repeated subsequently.

The State pays half of the value of the slaughtered cattle whenever the meat cannot be used for consumption, on condition that the owner has the rest of the animals inoculated with tuberculin.

The government pays 70 per cent. of the value of the animals dispatched according to its order in case the meat is unfit for consumption; if it can be used, the owner shall receive only 25 per cent. of the value of the animal.—W. C. S., in the January *Tijdschrift voor Veeartsenijkunde en Veeteelt.*

